# Isothiocyanates: cholinesterase inhibiting, antioxidant, and anti-inflammatory activity

**DOI:** 10.1080/14756366.2018.1442832

**Published:** 2018-03-07

**Authors:** Franko Burčul, Ivana Generalić Mekinić, Mila Radan, Patrick Rollin, Ivica Blažević

**Affiliations:** a Department of Analytical Chemistry, Faculty of Chemistry and Technology, University of Split, Split, Croatia;; b Department of Food Technology and Biotechnology, Faculty of Chemistry and Technology, University of Split, Split, Croatia;; c Department of Biochemistry, Faculty of Chemistry and Technology, University of Split, Split, Croatia;; d ICOA, UMR 7311, Université d’Orléans et CNRS, Orléans, France;; e Department of Organic Chemistry, Faculty of Chemistry and Technology, University of Split, Split, Croatia

**Keywords:** Isothiocyanates, antioxidation, cholinesterase inhibition, anti-inflammatory activity

## Abstract

Finding a new type of cholinesterase inhibitor that would overcome the brain availability and pharmacokinetic parameters or hepatotoxic liability has been a focus of investigations dealing with the treatment of Alzheimer’s disease. Isothiocyanates have not been previously investigated as potential cholinesterase inhibitors. These compounds can be naturally produced from their glucosinolate precursors, secondary metabolites widely distributed in our daily *Brassica* vegetables. Among 11 tested compounds, phenyl isothiocyanate and its derivatives showed the most promising inhibitory activity. 2-Methoxyphenyl ITC showed best inhibition on acetylcholinesterase with IC_50_ of 0.57 mM, while 3-methoxyphenyl ITC showed the best inhibition on butyrylcholinesterase having 49.2% at 1.14 mM. Assessment of the antioxidant efficacy using different methods led to a similar conclusion. The anti-inflammatory activity was also tested using human COX-2 enzyme, ranking phenyl isothiocyanate, and 3-methoxyphenyl isothiocyanate as most active, with ∼99% inhibition at 50 μM.

## Introduction

Alzheimer’s disease is the most common neurodegenerative disorder in western societies, mostly affecting elderly population. The most prominent symptom includes decrease in cognitive function, which in turn leads to changes in the behavioural patterns of an individual. Both acetylcholinesterase and butyrylcholinesterase still represent the only pharmacotherapy able to affect the increase of the acetylcholine neurotransmitter in the brain^1–3^. Essential oils, containing small lipophilic molecules, such as terpenes, terpenoids, phenylpropanoids, and others, were increasingly studied lately since they can readily cross the blood-brain barrier^4^. Interestingly, there are no reports whatsoever on the so called “mustard oils”, the major constituents of which are isothiocyanates (ITCs), known for their diversified and generally marked bioactivity^5^. Mustard oils stand as bio-markers of *Brassica* vegetables which encompass many species used in daily diets (cabbage, horseradish, cauliflower, turnip, radish, cress, etc.).

Oxidative stress is most commonly associated with the development of many diseases, including neurodegenerative disorders^6^. Different studies have mentioned ITCs as antioxidants, particularly in relation with induction of phase II enzymes, though a limited number of them have shown direct antioxidant behaviour^7^. The antioxidant potentials of volatile methylsulfanylalkyl ITCs such as erucin and raphasatin were revealed by effective quenching of hydrogen peroxide and organic hydroperoxides^8^^,^^9^ which are commonly classified as reactive oxygen species (ROS) and are important contributors to oxidative stress *in vivo*.

Antioxidant and anti-inflammatory effects of ITCs are likely mediated *via* the activation of Nrf2 and inhibition of NF-κB^10^^,^^11^. The anti-inflammatory properties of allyl ITC and other plant-derived ITCs have been previously reported^10^^,^^12^^,^^13^. After oral administration, allyl ITC is distributed in the tissues as well as in the brain of rats and mice^14^. Additionally, it was reported that ITCs protect the blood-brain barrier from oxidative stress-induced injury^15^. Overproduction of PGE-2 and increased COX-2 activity are frequently observed in a variety of inflammation-associated disorders, such as Alzheimer's disease, Parkinson's disease, certain kinds of cancers, and cardiovascular problems^10^^,^^16^^,^^17^.

In the present study, 11 commercially available ITCs were tested for their cholinesterase inhibitory (using Ellman’s method), and antioxidant (using DPPH, ORAC, Briggs-Rauscher, Rancimat, and FRAP method) activities as well as anti-inflammatory activity via PGE-2 synthesis suppression through COX-2 inhibition.

## Materials and methods

All reagents and solvents used were of analytical grade. Isothiocyanates **1–11** were purchased from Sigma-Aldrich GmbH (Steinheim, Germany). Acetylcholinesterase (AChE, from *Electrophorus electricus* – electric eel, type V-S), butyrylcholinesterase (BChE, from equine serum), acetylthiocholine iodide (ATChI), butyrylthiocholine iodide (BTChI), and 5,5-dithiobis(2-nitrobenzoic acid) (DTNB, Ellman’s reagent), were also purchased from Sigma-Aldrich. The human COX-2 inhibitor screening assay kit was purchased from Cayman Chemical (Item № 701080, Ann Arbor, MI). Indomethacin (purity 99%) was obtained from Fluka (Buchs, Switzerland). Absorbance and fluorescence measurements were performed on a Synergy HTX S1LFA multi-mode microplate reader (BioTek Instruments, Inc., Winooski, VT).

### Antioxidant activity

Assessment of the antioxidant activity was carried out using five methods: scavenging ability of 2,2-diphenyl-1-picrylhydrazyl radical (DPPH˙), Oxygen Radical Absorbance Capacity (ORAC), Briggs–Rauscher (BR) oscillating reaction, Rancimat assay and Ferric Reducing/Antioxidant Power (FRAP) method.

#### DPPH assay

DPPH scavenging ability of the samples was measured according to a recently reported procedure^18^. The results for free radical scavenging activities of the samples are expressed as IC_50_ values (where possible) and inhibition percentages of DPPH radical (% inhibition).

#### ORAC assay

ORAC assay was performed according to the slightly modified procedure described by Prior et al.^19^. The volumes were modified so as to perform in 96-well microplates and the reaction was followed during 80 min. The results are expressed as mM of Trolox Equivalents (mM TE).

#### Briggs–Rauscher assay

ITCs ability to stop the Briggs–Rauscher (BR) oscillations was assayed as described previously^20^. BR assay is based on the fact that the addition of antioxidant in oscillating BR system (visually detectable as fast colour change between blue → yellow → colourless) results in immediate quenching of the oscillations (visually = no colour). The quenching time linearly depends on the type and concentration of the added antioxidant. The oscillations in the BR assays were followed spectrophotometrically at 620 nm and the results are expressed as the inhibition time (in seconds).

#### Rancimat assay (oxidation stability testing)

Fish oil was investigated using a Rancimat 743 (Metrohm, Herisau, Switzerland) instrument in order to monitor the progress of accelerated oxidation. The fish oil samples (3 g) were exposed to a temperature of 120 °C (Δ*T* = 1.4 °C) and a constant air flow of 20 L/h. The conductivity was measured as a function of time and the results are expressed as Protection Factor (PF), calculated according to the equation PF = sample induction time/control induction time^21^.

#### FRAP assay

The reducing potential of ITCs was measured as described by Benzie and Strain^22^. In this assay, antioxidants are evaluated as reducing agents of Fe^3+^ to Fe^2+^, which undergoes chelation by 2,4,6-tris(2-pyridyl)-*s*-triazine (TPTZ) to form a Fe^2+^-TPTZ complex absorbing at 593 nm.

### Acetylcholinesterase/butyrylcholinesterase inhibitory activity

AChE/BChE inhibitory activity measurements were carried out by a slightly modified Ellman assay as described before for AChE inhibitory activity^23^. A typical run consisted of 180 μL of phosphate buffer (0.1 M, pH 8), 10 μL of DTNB (at a final concentration of 0.3 mM prepared in 0.1 M phosphate buffer pH 7 with 0.12 M sodium bicarbonate added for stability), 10 μL of sample solution (dissolved in EtOH), and 10 μL of AChE/BChE solution (with final concentration 0.03 U/mL). Reactants were mixed in a cuvette and reaction was initialised by adding 10 μL of acetylthiocholine iodide/butyrylthiocholine iodide (ATChI/BTChI, to reach a final concentration of 0.5 mM). As negative control, EtOH was used instead of sample solution. Non-enzymatic hydrolysis was also monitored by measurement of two blank runs for each run. In short: in the first blank, the AChE/BChE, respectively, was replaced by equivalent buffer amount and in second blank, the ATChI/BTChI, respectively, was replaced by equivalent buffer amount. All spectrophotometric measurements were performed at 405 nm and at room temperature for 6 min periods. The results are expressed as percentage inhibition of enzyme activity.

### Anti-inflammatory activity

To analyse the inhibition of the COX-2 enzyme activity mediated by the selected ITCs, the COX-2 (human) inhibitor screening assay kit from Cayman was used. In summary, the COX-2 enzyme was incubated with the samples (having final concentration in the assay of 50 μM, in 96% ethanol) for 10 min at 37 °C and subsequently the reaction was initiated by adding arachidonic acid and incubating the mixture for 2 min at 37 °C. The background tubes correspond to inactivated COX-2 enzyme obtained after keeping the tubes containing enzymes in boiling water for 3 min along with sample solvent. Enzyme catalysis was blocked by adding saturated stannous chloride solution. Prostaglandin (PGF2α) release was quantified using ELISA at 412 nm. Indomethacin solution (having final concentration in the assay of 10 μM, in 96% ethanol) was used as a positive control.

## Results and discussion

**Figure 1.**
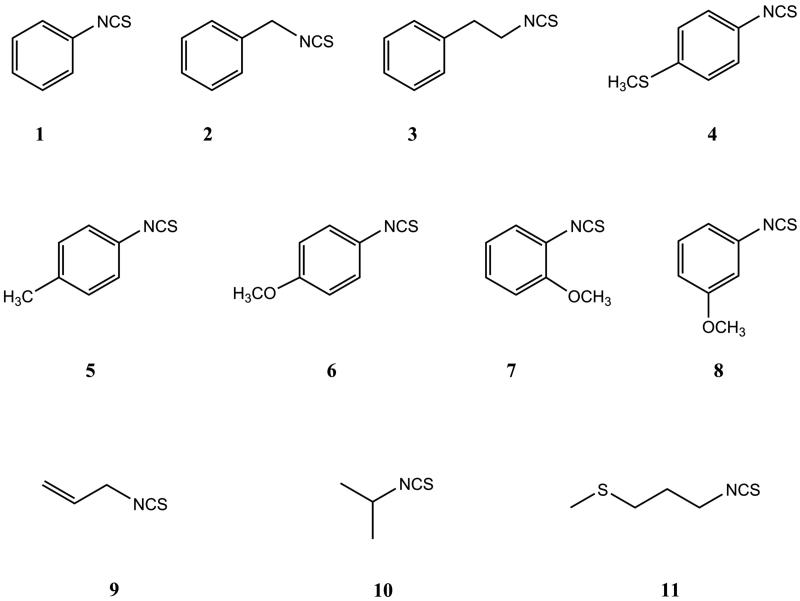
Structures of the isothiocyanates (ITCs) used in the current study: **1** – phenyl ITC, **2** – benzyl ITC, **3** – 2-phenylethyl ITC, **4** – 4-methylsulfanylphenyl ITC, **5** – 4-methylphenyl ITC, **6** – 4-methoxyphenyl ITC, **7** – 2-methoxyphenyl ITC, **8** – 3-methoxyphenyl ITC, **9** – allyl ITC, **10** – isopropyl ITC and **11** – 3-(methylsulfanyl)propyl ITC.

Structures of isothiocyanates (ITCs) tested on different biological activities are shown in Figure 1.

### Antioxidant activity

#### Free radical scavenging activity

The free radical scavenging potential of ITCs was determined by DPPH, ORAC, and BR antioxidant assays (Table 1). For practical purposes, the concentrations of the investigated compounds tested by DPPH method are given as stock solutions and should be divided by a factor of 21 to obtain the final concentration in the system (Table 1), and are discussed as such in the text ahead. The half-maximal DPPH radical scavenging concentration (IC_50_) was calculated for phenyl ITC (**1**), 3-methoxyphenyl ITC (**8**), 4-methoxyphenyl ITC (**6**), 4-methylphenyl ITC (**5**), and 2-methoxyphenyl ITC (**7**) having IC_50_ of 1.08, 1.16, 1.25, 1.45 and 3.90 mM, respectively. 4-Methylsulfanylphenyl ITC (**4**) had to be tested at lower concentration due to turbidity problems, with 1.19 mM (final concentration) showing 9.9% of DPPH inhibition. Montaut et al.^24^ reported 4-hydroxybenzyl ITC to have IC_50_ of 9.43 mM. This compound, having OH group as a strongly activating substituent on the aromatic ring, showed a much weaker activity than phenyl ITC (**1**). 3-Methoxyphenyl ITC (**8**), 4-methoxyphenyl ITC (**6**), 4-methylphenyl ITC (**5**), and 2-methoxyphenyl ITC (**7**), bearing a substituent on the aryl ring, also exhibited a decrease in the DPPH activity, thus indicating a negative effect of substitution. When the ITC group was not directly attached on the aryl ring like in benzyl ITC (**2**) and 2-phenylethyl ITC (**3**), the free radical scavenging ability also rapidly decreased.

**Table 1.** Antioxidant properties of isothiocyanates (ITCs).

Compound name	DPPH		ORAC (mM TE)^b^	BR (s)^c^	Rancimat (PF)^c^
	IC_50_ (mM)	Inhibition (%)^a^			
Phenyl ITC (**1**)	1.08 ± 0.01	88.4 ± 1.8	26.3 ± 0.1	n.d.	2.5
Benzyl ITC (**2**)	–	9.9 ± 0.4	4.0 ± 1.6	n.d.	1.5
2-Phenylethyl ITC (**3**)	–	n.d.	4.8 ± 0.5	n.d.	1.3
4-Methylsulfanylphenyl ITC (**4**)	–	9.9 ± 0.6	32.3 ± 3.6	65 ± 2	1.1
4-Methylphenyl ITC (**5**)	1.45 ± 0.02	80.6 ± 0.6	16.3 ± 5.0	n.d.	1.0
4-Methoxyphenyl ITC (**6**)	1.25 ± 0.02	80.4 ± 0.9	11.7 ± 0.5	>1 h^d^	1.0
2-Methoxyphenyl ITC (**7**)	3.90 ± 0.03	60.9 ± 0.4	1.6 ± 0.1	n.d.	1.7
3-Methoxyphenyl ITC (**8**)	1.16 ± 0.03	84.8 ± 0.5	20.9 ± 0.9	10 ± 1	1.7
Allyl ITC (**9**)	–	10.1 ± 0.3	9.5 ± 1.6	430 ± 8	1.7
Isopropyl ITC (**10**)	–	4.2 ± 0.6	1.4 ± 0.5	n.d.	1.1
3-(Methylsulfanyl)propyl ITC (**11**)	–	12.6 ± 1.3	20.3 ± 3.8	23 ± 3	1.6

TE: trolox equivalents; PF: protection factor (control PF = 1.00); –, not determined; n.d.: not detected.

All compounds were tested at a concentration of: ^a^4.76 mM, except for **4** which was at 1.19 mM due to turbidity; ^b^1.56 μM; and ^c^3.23 mM.

^d^For 3.23, 1.61, 1.29 and 0.81 mM did not start to oscillate after 1 h. For 0.65 and 0.48 mM of added compound oscillation lasted 470 ± 11 and 38 ± 5 s, respectively. Measurement was effected visually for 0.81 mM and the reaction started to oscillate after 9180 ± 15 s.

**Figure 2.**
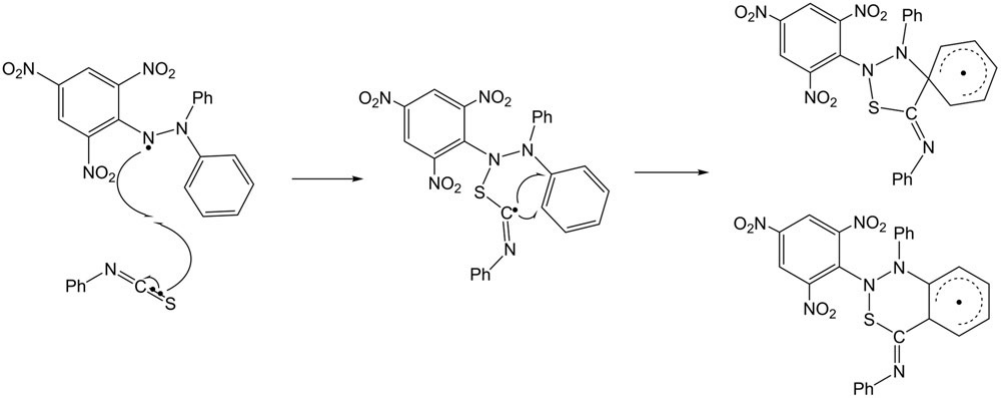
Proposed mechanism for the reaction of phenyl ITC with the DPPH radical.

Benati et al.^25–27^ investigated the attack of aryl radicals on ITCs, highlighting the variation of electrophilicity of the radical depending on substituents. Thus, electron-withdrawing substituents would favour the nucleophilic *S*-addition of the ITC, hence increasing the overall efficiency of the cascade reaction. In connection with the results of Benati et al.^25–27^, one may hypothesise that the S-atom of ITCs reacts with the DPPH radical, to produce an α-(arylsulfanyl)imidoyl radical that can gain stabilisation through interference with one of the phenyl rings of DPPH (Figure 2). However, it was observed that aryl ITCs (**5**–**8**) bearing electron-donating groups showed a decrease in free radical scavenging ability when compared to “group free” phenyl ITC (**1**).

3-(Methylsulfanyl)propyl ITC (**11**), allyl ITC (**9**), and isopropyl ITC (**10**) were also tested and they were found to be very inefficient scavengers at 4.76 mM having 12.6, 10.1 and 4.2%, respectively. Montaut et al.^24^ reported 4-(methylsulfanyl)butyl ITC (erucin) to be also very ineffective, having IC_50_ of 57.1 mM. 4-(Methylsulfanyl)but-3-enyl ITC (raphasatin) was reported to have a high reactivity toward the DPPH radical, presumably due to hydrogen atom abstraction either in allylic position, or in alpha to the –NCS group or both^9^. 4-(Methylsulfinyl)butyl ITC (sulforaphane) also showed scavenging activity of DPPH radical with IC_50_ of 3.8 mM^28^.

Alkyl ITCs bearing a sulfanyl group (like erucin, raphasatin and ibervirin **11**) in the side-chain and arylalkyl ITCs (like **2** and **3**) are expected to react with radicals by H-atom transfer from the methylene group neighbouring the S-atom or the phenyl ring^7^, contrary to the cases described by Benati et al.^25^^,^^26^, for which such methylene groups are missing. When tested by the DPPH method, ITCs **2**, **3** and **11** showed very insignificant antiradical activity. Similarly, ITC **3** was reported by Sarkar et al.^29^ to be a very inefficient free radical scavenger.

ORAC assay is a method that combines both inhibition percentage and inhibition time of the reactive species action by antioxidants into a single quantity^30^. It reflects classical radical chain breaking antioxidant activity by H atom transfer and it measures antioxidant inhibition of peroxyl radical induced oxidations^31^. Although Valgimigli and Iori^7^ reported that ITCs do not express chain-breaking activity, our study produced contrasting results. Samples were tested using ORAC assay at several concentrations (results not shown) similar to those of DPPH, but they had to be diluted due to the high sensitivity of the ORAC method. Results are presented in Table 1 with all the compounds measured at the final concentration of 1.56 µM. According to the present results the highest activity of 32 mM TE was detected for 4-methylsulfanylphenyl ITC (**4**). The activity of this compound was not detected by DPPH method due to turbidity problems, while the ORAC method circumvents turbidity problems since it is based on measurement of fluorescence. Direct connection of the –NCS group to the aromatic ring resulted in good activity of the ITCs, as previously observed, while in the case of compounds **2** and **3** a significant decrease in the activity was detected. The lowest activity was found for 2-methoxyphenyl ITC (**7**) and isopropyl ITC (**10**), as well as for arylalkyl ITCs **2** and **3**. Among methoxyphenyl ITCs (**6**, **7** and **8**) the highest free radical scavenging activity was detected for 3-methoxyphenyl ITC (**8**) while among alkyl ITCs bearing a sulfanyl group, a remarkable activity was observed for 3-(methylsulfanyl)propyl ITC (**11**) (20.3 mM TE).

The Briggs–Rauscher assay gave more or less no activity for any of the ITCs at the highest tested concentration except for 4-methoxyphenyl ITC (**6**) that prolonged oxidation for 9180 s at final concentration of 0.81 mM. However, the *ortho-* (**7**) and *meta*- (**8**) regioisomers of **6** did not exhibit activity at four times higher concentration. Chen et al.^32^ reported comparable observations when investigating cyclohexanedione isomers: the 1,3-dione showed inhibition time, whereas the 1,4-dione did not.

#### Reducing activity

ITCs were also tested using FRAP assay, which is presumed to estimate the “total antioxidant power”. The relevant chemical reaction of the FRAP method represents the “total reducing power” as it involves a single electron reaction between Fe(TPTZ)_2_ (III) and any species able to reduce it to Fe(TPTZ)_2_ (II), making this species an antioxidant^31^. In our study, none of the ITCs tested showed any reductive capacity.

#### Oxidative stability testing

The antioxidant activity of ITCs was also tested by means of a Rancimat apparatus, using a lipophilic system at 120 °C with an air flow rate of 20 L/h. The results are expressed as protection factors (PF), taking PF = 1 for the control sample. The best activity was found for phenyl ITC (**1**) with PF = 2.5 at 3.23 mM. For ITCs in which the –NCS group is not directly attached to the aromatic ring, the activity is decreasing (compared to **1**) as seen for compounds **2** (PF = 1.5) and **3** (PF = 1.3) at the same concentration. Some of the ITCs – either alkyl or aryl – exhibited activities close to that of **2**, i.e. allyl ITC (**9**), 3-methoxyphenyl ITC (**8**), 2-methoxyphenyl ITC (**7**), with PF = 1.7, and 3-(methylsulfanyl)propyl ITC (**11**) with PF = 1.6 at 3.23 mM. 4-Methylsulfanylphenyl ITC (**4**), 4-methylphenyl ITC (**5**), 4-methoxyphenyl ITC (**6**), and isopropyl ITC (**10**) did not show any protective effect.

### Cholinesterase inhibition

To the best of our knowledge, the present paper reports the first investigation on cholinesterase (ChE) inhibitory activity of ITCs. The inhibition of acetylcholinesterase (AChE) and butyrylcholinesterase (BChE) was assessed for 6 aromatic, 2 arylaliphatic, and 3 aliphatic ITCs and results are reported in Table 2. Generally, aromatic and arylaliphatic ITCs showed better activity than aliphatic ITCs (except for ITC **4**, due to the turbidity problems at higher concentrations). The best activity was shown by 3-methoxyphenyl ITC (**8**) with 61.4% of AChE inhibition at 1.14 mM. *Para*- (**6**) and *ortho*- (**7**) isomers of ITC **8** also showed relatively good activity with 30.4 and 57.0%, respectively, at the same concentration. The inhibitory activity of 4-methylphenyl ITC (**5**) on AChE was comparable to that of **7** and **8** with 58.4% at 1.14 mM. Electron donating groups (–OMe, –Me) seem to contribute to the increase of inhibitory activity when compared to phenyl ITC (**1**) which showed only 17.9% at the same concentration. In the case of arylaliphatic ITCs **2** and **3**, the inhibitory activity was also relatively good with 37.2 and 48.1%, respectively, at 1.14 mM.

**Table 2.** Cholinesterase and COX-2 inhibition by isothiocyanates (ITCs).

Compound name	AChE		BChE	COX-2
	IC_50_ (mM)	Inhibition (%)^a^	Inhibition (%)^a^	Inhibition (%)^b^
Phenyl ITC (**1**)	–	17.9 ± 0.1	42.1 ± 0.7	98.9 ± 0.3
Benzyl ITC (**2**)	–	37.2 ± 0.1	10.3 ± 0.5	n.d.
2-Phenylethyl ITC (**3**)	–	48.1 ± 0.7	n.d.	17.8 ± 0.6
4-Methylsulfanylphenyl ITC (**4**)	–	14.3 ± 0.6	9.3 ± 0.9	15.5 ± 0.2
4-Methylphenyl ITC (**5**)	0.93	58.4 ± 1.1	40.7 ± 0.7	13.6 ± 0.8
4-Methoxyphenyl ITC (**6**)	–	30.4 ± 0.4	17.9 ± 0.3	n.d.
2-Methoxyphenyl ITC (**7**)	0.57	57.0 ± 1.5	19.5 ± 0.1	99.0 ± 0.7
3-Methoxyphenyl ITC (**8**)	1.00	61.4 ± 1.6	49.2 ± 0.6	n.d.
Allyl ITC (**9**)	–	n.d.	n.d.	19.5 ± 0.1
Isopropyl ITC (**10**)	–	3.2 ± 0.3	n.d.	9.4 ± 0.5
3-(Methylsulfanyl)propyl ITC (**11**)	–	4.4 ± 0.7	15.4 ± 0.3	48.0 ± 0.3

–: not determined; n.d.: not detected.

All compounds were tested at a concentration of: ^a^1.14 mM, except for **4** which was at 0.19 mM due to turbidity; ^b^final concentration was 50 μM, while the concentration of the referent compound – indomethacin was 10 μM showing 98.9 ± 0.2% inhibition.

In general, all tested compounds showed lower inhibitory activity towards BChE, except for compounds **1** and **11**. As observed in the case of AChE, the inhibitory activity of the aromatic ITC **8** towards BChE was shown to be the best with 49.2% at 1.14 mM. Closely related aryl ITCs **1** and **5** also showed notable activity with 42.1 and 40.7%, respectively, at the same concentration. In comparison to **1**, the inhibitory activity towards BChE is similar (**5**, **8**) or inferior when substituents are present on the aromatic ring (**6**, **7**), or when the –NCS group is not directly attached to the aromatic ring (**2**). Aliphatic ITCs **9**–**11** showed very weak activity or no inhibition at all tested concentrations on both enzymes.

### Anti-inflammatory activity

Results for the inhibition of prostaglandin biosynthesis, by ITCs as determined using COX-2 assay, are given in Table 2 and are expressed as % of inhibition. Indomethacin was used as a positive control having 98.9% inhibition at the final concentration of 10 μM. Among the 11 tested ITCs, phenyl ITC (**1**), 2-methoxyphenyl ITC (**7**), and 3-(methylsulfanyl)propyl ITC (**11**) exhibited significant inhibitory activity of COX-2 enzyme with 98.9, 99 and 48%, respectively, at final concentration of 50 μM. At the same concentration, ITCs **2**, **6**, and **8** did not show any inhibitory activity on prostaglandin production. On the other hand, ITCs **9**, **3**, **4**, **5** and **10** showed moderate to low inhibitory activity having 19.5, 17.8, 15.5, 13.6 and 9.4%, respectively, at final concentration of 50 μM. The effective inhibitory activity of **1** is in accordance with previously reported results on suppression of PGE_2_ synthesis^11^. Bhattacharya et al.^33^ also reported that **9**, the most common and most studied naturally occurring ITC, showed no expected inhibitory activity against COX-2. However, studies of *in vitro* ITC inhibitory activity on COX-2 enzyme are scarce. The data presented in Table 2 indicate that inhibitory activity of the tested ITCs is quite specific. It seems that a slight change in the ITC structure can have a significant impact on its inhibitory potential, i.e. **7** showing strong inhibitory activity contrary to its regioisomers **6** and **8** which proved inactive at the tested concentration. The effective inhibition of prostaglandin synthesis by ITCs **1**, **7** and **11** calls for further investigation of their effects on COX-2 protein and mRNA expression.

## Conclusion

Herein, we tested 11 ITCs for their biological activity (i.e. antioxidative, anti-cholinesterase, and anti-inflammatory). Generally, among the tested ITCs, it can be suggested that aryl ITCs show the most promising potential. These results can be used as a basis for future investigation of other phenyl ITC derivatives bearing electron-donating and -withdrawing groups in order to better understand the influence of diversified substituents towards different biological activities.
